# Low genetic diversity and strong immunogenicity within the apical membrane antigen-1 of *plasmodium ovale* spp. imported from africa to china

**DOI:** 10.1016/j.actatropica.2020.105591

**Published:** 2020-10

**Authors:** Yao Lei, Feihu Shen, Haimeng Zhu, Laicheng Zhu, Ruilin Chu, Jianxia Tang, Wenxi Yao, Guoding Zhu, Dengxin Zhang, Jun Cao, Yang Cheng

**Affiliations:** a Laboratory of Pathogen Infection and Immunity, Department of Public Health and Preventive Medicine; Wuxi School of Medicine, Jiangnan University, Wuxi, Jiangsu, China; b Department of Anesthesiology, Affiliated Hospital of Jiangnan University, Wuxi, Jiangsu, China; c National Health Commission Key Laboratory of Parasitic Disease Control and Prevention, Jiangsu Provincial Key Laboratory on Parasite and Vector Control Technology, Jiangsu Institute of Parasite Diseases, Wuxi, Jiangsu, People’ s Republic of China

**Keywords:** Immunogenicity, Plasmodium ovale, Apical membrane antigen-1, Cross-reactivity, AMA-1, Apical membrane antigen-1, rPoAMA-1, recombinant PoAMA-1, PCR, Polymerase chain reaction, IPTG, Isopropyl β-D-thiogalactoside, aa, amino acid, RT, Room temperature, E. coli, Escherichia coli, TBST, Tris-Buffered Saline Tween-20, ELISA, Enzyme-linked immunosorbent assays, TMB, 3,3′,5,5′-Tetramethylbenzidine, AI, Avidity index, RPMI, Roswell Park Memorial Institute

## Abstract

Malaria is still an important challenge for global public health because of its extensive mortality and morbidity. *Plasmodium ovale* is mainly distributed in tropical regions of Africa and Asia. it includes two distinct *ovale* malaria species, which are *P. ovale curtisi* and *P. ovale wallikeri*. Apical membrane antigen-1 (AMA-1) is an asexual blood-stage protein which is essential for *Plasmodium*. Thus far, no study on gene polymorphism and immunogenicity of *P. ovale* AMA-1 (PoAMA-1) has been conducted. Amplified *poama1* gene products from 14 *P ovale curtisi* samples and 12 *P ovale wallikeri* samples imported from Africa to Jiangsu Province, China were sequenced and their polymorphisms were analyzed. We expressed recombinant PoAMA-1 (rPoAMA-1, 53 kDa) proteins in an *E. coli* expression system and evaluated immune responses against the rPoAMA-1 in BALB/c mice. We identified a synonymous mutation in nucleotide position 333 of the *pocama-1* gene and *powama-1* did not reveal any variation. The humoral and cellular immune responses to rPoAMA-1 were evaluated using enzyme-linked immunosorbent assay (ELISA) and flow cytometry. rPoAMA-1-immunized mice produced specific antibodies as verified by immunoblotting. The rPoAMA-1 induced high antibody titers (1: 640,000), and had high avidity indexes (an average of 78.63% and 83.40%). The antibodies also recognized the native proteins, namely, crude antigen from blood stages. Cross-reactivity between rPocAMA-1 and rPowAMA-1 was observed. Moreover, rPoAMA-1 s induced interferon (IFN)-gamma-secreting cells in mice and increased lymphocyte proliferation response. Low genetic diversity was observed in poama-1 from the Jiangsu Province imported malaria cases, and further studies conclusively showed its strong immunogenicity. Significant cross-reactivity was found between rPocAMA-1 and rPowAMA-1, suggesting that a single PoAMA-1 antigen could be used to diagnose *P. ovale curtisi* or *P. ovale wallikeri* patient simultaneously. However, further evaluation needs to be carried out to validate the potential and limitations of PoAMA-1 as a candidate vaccine.

## Highlights

• Low genetic diversity of *poama-1* gene which is a neglected malaria parasite.
• rPoAMA-1 protein can induce strong immunogenicity in mice.
• Cross-reactivity between rPocAMA-1 and rPowAMA-1 antigen is present.

## 1 Introduction

Malaria has been a major global human health problem throughout history. According to the World Malaria Report in 2019, approximately 228 million cases of malaria exist worldwide (WHO, 2019). Malaria causes widespread morbidity and mortality and is still a major challenge to global health. *Plasmodium falciparum* is the most common malaria parasite in Africa (WHO, 2019). However, *P. vivax* is the major parasite in the American region, accounting for 74.1% of malaria cases. *P. ovale*, a type of benign recurrent malaria, is not highly studied compared with *P. falciparum* and *P. vivax. P. ovale* is mainly distributed in sub-Saharan Africa and in the western Pacific islands, occasionally in Southeast Asia and India (Ansari et al., 2016; Mueller et al., 2007). *P. ovale* is named after the typical elliptical appearance of infected erythrocytes. *P. ovale* and *P. vivax* share similar morphologies and phenotypes (Collins et al., 2005). However, *P. ovale* often occurs in mixed infections with other malaria parasite species, most commonly with *P. falciparum* and *P. malariae*. In addition, due to low parasitemias and co-infecting malaria parasite species. Its typical hyperparasitaemia is difficult to diagnose by optical microscopy; thus, morbidity and mortality due to *P. ovale* are underestimated (Dinko et al., 2013), which may become an obstacle to the elimination of malaria. Two morphologically identical but different species, *P. ovale curtisi* and *P. ovale wallikeri*, were identified by genetic marker typing (Sutherland et al., 2010; Tachibana et al., 2002).

The genome of the *Plasmodium* parasite encodes more than 5000 proteins, but function is known only for few proteins. *Plasmodium* AMA-1 is relatively conserved across species and has homologs in *Toxoplasma gondii* and *Babesia bovis*, which are present in micronemes and then on the surface of mature merozoites during the blood stage of *Plasmodium* (Gentil et al., 2010; Li et al., 2003; Remarque et al., 2008). During merozoite invasion of red blood cells, AMA-1 (formerly known as Pf83 and Pk66) plays a key role by interacting with Rhoptry Neck Protein 2 (RON2) (Cao et al., 2009; Lamarque et al., 2011). Attempts to generate stable AMA-1 knockouts in several *Plasmodium* species have failed, thereby suggesting that AMA-1 is critical for parasite growth in the blood stage (Triglia et al., 2000). AMA-1 is a major malaria vaccine candidate. Immune response to *P. falciparum* AMA-1 was confirmed. This finding demonstrates that anti-AMA-1 antibodies mediate protection and validate the immune responses against AMA-1 *in vitro* models that demonstrated significant parasite inhibition (Hodder et al., 2001; Remarque et al., 2008, 2008). Studies of recombinant proteins on the basis of the ectodomain sequence of AMA-1 of different *Plasmodium* species have further provided strong evidence that AMA-1 can be used as an antigen for subunit malaria vaccines (Múfalo et al., 2008). However, due to the extensive polymorphism of AMA-1, its development as a malaria vaccine antigen is limited (Duan et al., 2008).

From 2011 to 2014, 1268 cases of malaria were reported in Jiangsu Province. Although the imported malaria cases were mainly caused by *P. falciparum*, the number of *ovale* and *malariae* malaria cases increased from yearly (Cao et al., 2016). The increase in *ovale* malaria has raised the potential risk of reintroduction of malaria in Jiangsu Province. However, few studies on the importation of *ovale* malaria are available, and the research on *P. ovale* is mainly a clinical observation and case report (Zhou et al., 2019). Studies on the importance of *P. ovale* cases in China are few, and data on the sequence diversity and immunogenicity of PoAMA-1 are limited. Therefore, we analyzed the genetic polymorphism of *poama1* from imported *P. ovale* malaria cases in Jiangsu Province to evaluate its immunogenicity.

## 2 Materials and methods

### 2.1 *P. ovale*-infected human blood samples

*P. ovale curtisi* and *P. ovale wallikeri* samples were obtained from local hospitals in the Jiangsu Province in China between 2012 and 2016 from febrile patients who had recently returned from working in tropical regions of sub-Saharan Africa, endemic for malaria (Chu et al., 2018). Identification of the isolates was confirmed by polymerase chain reaction (PCR) analysis, and *Plasmodium* species were distinguished by real-time TaqMan PCR (Cao et al., 2016). *P. ovale curtisi* (14 cases) and *P. ovale wallikeri* (12 cases) were identified (Additional file 1: Table S1).

### 2.2 Cloning and sequence alignment

Twenty-six cases of *P. ovale* spp. malaria imported from Africa were identified and selected for the PCR (Eppendorf, Hamburg, Germany) amplification of full-length sequences for *poama-1* (1689 bp). The *poama-1* primers used were *pocama-1*-Forward (5′-ATG AAG AAA ATA TAC-3′) and *pocama-1*-Reverse (5′-TTA ATA TGG CTT TTC-3′) and *powama-1*-Forward (5′-ATG AAG AAA ATA TAC-3′) and *powama-1*-Reverse (5′- ATA TGG CTT TTC CAT-3′). The PCR amplification conditions used are as follows: an initial denaturation step at 95 °C for 3 min; 35 cycles at 95 °C for 15 s, 55 °C for 30 s, and 72 °C for 30 s; a final extension step at 72 °C for 5 min. The amplified products were analyzed by agarose gel (1%) electrophoresis and visualized under ultraviolet transilluminator (Bio-Rad ChemiDoc MP, Hercules, USA). Then, the PCR products were ligated to the pUC57 vector and sequenced using pUC57 universal primers (M13R and M13F) by GENEWIZ (Suzhou, China).

The *pocama-1* (PlasmoDB, PocGH01_09039800) and *powama-1* (GenBank accession number, SBT36045.1) sequences were used as templates for sequencing and polymorphism analysis of the PoAMA-1 sequence (26 samples) aligned using GeneDoc 2.7.0 (Nicholas and Nicholas, 1997). We aligned the amino acid sequence of AMA-1 using GeneDoc 2.7.0 and NCBI blast, and then modelled AMA-1 protein (PF3D7_1,133,400, PVX_092275, POVCU2_0023170, POVWA1_028140, PlasmoDB) tertiary using SWISS-MODEL (https://www.swissmodel.expasy.org/).

### 2.3 Expression and purification of rPoAMA-1 s

rPoAMA-1 s were designed from the gene sequences of *pocama-1* (GenBank accession numbers: MT346601) and *powamam-1* (GenBank accession numbers: MT346602). The *poama-1* genes (N-terminal) were amplified using the genomic DNA of *P. ovale* isolates and cloned into the pET-30a (+) vector with the in-fusion cloning primers Poc-F (5′-ATG GCT GAT ATC GGA TCC ATG AAG AAA ATA TAC ATC T-3′), Poc-R (5′- GTG CTC GAG GGA AGA TGT AGT ATA TTT-3′), and Pow-R (5′- GTG CTC GAG TCT ACA TCT GGA TGA-3′). The vector sequences and *Xho*I and *Bam*HI sites are represented by the lowercase characters. BL21(DE3) pLysS cells containing recombinant plasmid rPoAMA-1 were propagated in Luria Bertani (LB) broth containing kanamycin (50 µg/mL) at 16 °C with shaking until the optical density reached 0.4–0.6 at 600 nm (OD600). Isopropyl β-d-1-thiogalactopyranoside (IPTG, 0.01 mM) (TransGen Biotech, Beijing, China) was used to induce the culture and allowed it to grow for another 24 h. Proteins were purified by YouLong Biotech (Shanghai, China).

### 2.4 SDS-PAGE and immunoblot analysis

The purified rPoAMA-1 proteins were separated using 8% SDS-PAGE, and then stained by Coomassie brilliant blue. For the western blot analysis, rPoAMA-1 s were transferred onto a solid support polyvinylidene fluoride (PVDF, Millipore, Massachusetts, USA) membrane and blocked with 5% nonfat milk in Tris-Buffered Saline Tween-20 (TBST) for 2 h and then reacted with the anti-his tag rabbit monoclonal antibody (1: 1000 dilution) (CST, Boston, USA) overnight at 4 °C. Then, the membrane was incubated with a horseradish peroxidase (HRP)-labeled goat anti-rabbit antibody (1: 5000 dilution) (SouthernBiotech, Alabama, USA) for 1 h at 37 °C. The fluorescence signals were scanned on a gel imaging analysis system (Bio-Rad ChemiDoc MP, Hercules, USA) and analyzed with Image J software (Bio-Rad ChemiDoc MP, Hercules, USA).

### 2.5 Mouse immunizations

Twenty seven-week-old female BALB/c mice (Hengtai, Wuxi, China) were used for immunization (PocAMA-1-immunized group, PowAMA-1-immunized group, PBS group and normal group; *n* = 5 per group). Each mouse was intraperitoneally injected with 50 µg of rPocAMA-1, rPowAMA-1, or PBS diluted in PBS with Freund's complete adjuvant (Sigma, San Francisco, USA). Booster injections were administered four and seven weeks later using the same amount of PoAMA-1 with Freund's incomplete adjuvant (Sigma, San Francisco, USA). Serum was collected from the tails of the mice on the seventh and 14th day after the first and second immunizations. Mouse blood samples were collected two weeks after the second booster and these sera samples were stored at −80 °C.

### 2.6 Immunoblot analysis of *P. ovale* crude proteins

The enriched *P. ovale* parasites were purified by Percoll (Meilunbio, Dalian, China) gradient centrifugation, and then harvested and solubilized in Radio Immunoprecipitation Assay (RIPA) buffer (P0013B, Beyotime Biotechnology, Beijing, China) and incubated on ice for 30 min. The supernatant was collected by centrifugation at 15,000 g for 15 min. The *P. ovale* lysate was mixed with SDS sample buffer and boiled for 10 min, and the insoluble material was removed by centrifugation (15,000 g for 5 min). The boiled parasite lysate was run on an SDS-PAGE gel and transferred to PVDF membrane. The membrane was incubated with mouse anti-rPoAMA-1 (1: 100 dilution) serum or PBS-immunized serum followed by goat antimouse secondary IgG (1: 5000 dilution). The protein band was detected through the gel imaging analysis system (Bio-Rad ChemiDoc MP, Hercules, USA).

### 2.7 Determination of igg specificity

Specific antibody titers of the PoAMA-1-immunized mouse were analyzed using the mouse immune serum by enzyme-linked immunosorbent assay (ELISA). The rPoAMA-1 (1 μg/mL, sodium carbonate buffer) was used to coat 96-well plates and then placed overnight at 4 °C. To reduce nonspecific binding, the wells were blocked with 5% (w/v) non-fat milk in TBST and incubated at 37 °C for 2 h. The plates were washed three to four times with wash buffer (TBST), followed by the addition of serially diluted serum. After 2 h of incubation at 37 °C, the plates were washed with TBST and incubated for 1 h with HRP-conjugated goat anti-mouse antibody (dilution 1: 5000) at RT. Then, the plates were washed again and 3,3′,5,5′-Tetramethylbenzidine (TMB; Beyotime Biotechnology, Beijing, China) was added to the plates at RT for 1 min. The reaction was stopped by adding 50 µL/well 2 M sulfuric acid, and color intensity was determined at 450 nm using microplate reader. All samples were tested in repeats, and the mean absorbance was calculated.

### 2.8 Avidity index

Specific IgG avidity measurements were carried out by ELISA with a 6 M urea elution step (Sravanthi et al., 2015) using the ELISA procedure previously described. Indirect ELISA was slightly modified in accordance with the IgG specificity protocol. In summary, after the serum sample was added, the test group of the plate was incubated with 6 M urea for 15 min, whereas the control group was incubated without urea. Subsequently, the plate was washed and the secondary antibody was added and incubated for 1 h. The reaction of TMB was terminated by sulfuric acid, and the color intensity was analyzed at 450 nm. Urea can dissociate low affinity antigen-antibody complexes; thus, the avidity index (AI) was calculated by the following formula (Vemireddy et al., 2015):

AI = 100% × (antigen-antibody incubated with 6 M urea / antigen-antibody incubated without urea)

### 2.9 Cell isolation and preparation

The mice were killed by cervical dislocation, and the spleens were aseptically removed from mice and immediately placed in 3 mL of RPMI1640 (Thermo Fisher Scientific, Massachusetts, USA). Single cell suspensions were prepared by grinding the spleen through a pore mesh (40 µm) and washing with RPMI1640. To lyse the erythrocytes, samples were centrifuged (1500 × *g*, RT, 10 min), and then the pellets were mixed with 2 mL of red blood cell lysis buffer (Solarbio) at 37 °C for 3 min. PBS was added to stop lysis of erythrocytes, and then centrifuged again. Then the cells were resuspended and counted using a hemocytometer.

### 2.10 Determination of lymphocyte proliferation using the CCK8 assay

Lymphocytes from mice immunized with PoAMA-1 and PBS (5 × 10^5^ cells/well) were treated with 10 µL Concanavalin A (Con A: 2 μg/mL), 10 µL PocAMA-1 (5 µg/mL), or 10 µL PowAMA-1 (5 µg/mL) in 96-well flat bottom microtiter plates. Then, the plates were incubated for 72 h at 37 °C with 5% CO_2_. Cell proliferation was conducted by using the Cell Counting Kit-8 (CCK-8; Beyotime Biotechnology, Beijing, China) assay, and the stimulation index (SI) was calculated by the following formula (Karami et al., 2016):

SI = [(OD_562_ of stimulated cells − OD_562_ of unstimulated cells) / OD_562_ of unstimulated cells] × 100%.

### 2.11 Measurement of cytokine levels in mice by flow cytometry

To measure IFN-γ levels by stimulating splenocytes *in vitro*, splenocytes were cultured in 12-well plates containing proteins of rPoAMA-1. After 18 h, the cells were simultaneously mixed with various concentrations of phorbol 12-myristate 13-acetate (50 ng/mL PMA; Sigma), ionomycin (1 µg/mL; Solarbio), and brefeldin A (5 µg/mL; Solarbio) for 6 h at 37 °C with 5% CO_2_. The cells were washed twice with PBS. Subsequently, 50 µL PBS containing CD4–488 (1:200; BioLegend, California, USA) and CD8-APC (1:300; BioLegend, California, USA) were added to samples at 4 °C for 1 h, and then centrifuged (1500 × *g*, 10 min) and washed twice with PBS. Then, 100 µL of fixative (BioLegend, California, USA) was added, and the samples were incubated in a dark environment at 4 °C for 20 min. The samples were then washed with membrane washing buffer (BioLegend, California, USA), and 50 μL of IFN-r-PE (1:200; BioLegend, California, USA) diluted in transmembrane washing solution was added in a dark environment at 4 °C overnight. Finally, the samples were analyzed in BD Accuri C6 PLUS flow cytometry (BD, New Jersey, USA).

### 2.12 Statistical analysis

Graphs were generated using GraphPad Prism software (version 5.0). Statistical analyses of cytokine levels were conducted using SPSS 16.0 and student's *t-test* with probability (*P*) value < 0.05, indicating statistical significance. The phylogenetic tree of AMA-1 was constructed by the Neighbor-Joining (NJ) method, based on the *ama-1* nucleotide sequences from the PlasmoDB database. MEGA software (version 7.0) was used to align the *ama-1* sequences and graph the evolutionary relationships. The general time reversible (GTR) nucleotide model with a gamma (γ)-distribution model of among-site rate variation and a proportion of invariable sites (*i.e.* the GTR + γ + I substitution model) was determined using MEGA v.7.0 software.

## 3 Results

### 3.1 Phylogenetic and bioinformatics analysis of the poama-1 sequence

**Fig. 1:**
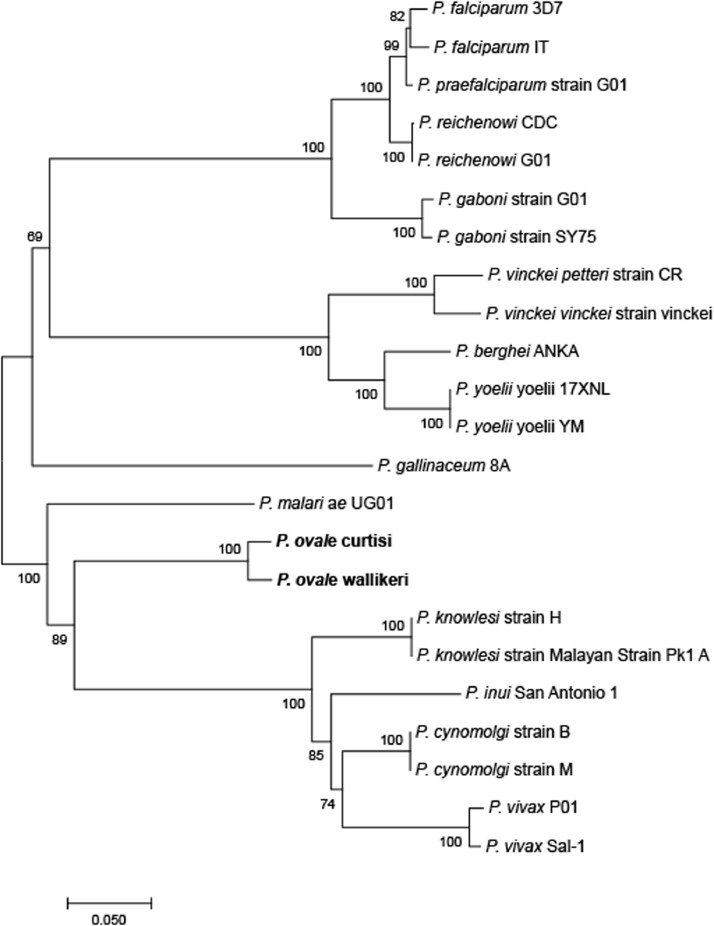
Phylogenetic relationship of *ama1* genes within ortholog *Plasmodium* species by using NJ method.

*poama-1* genes at full length (1–1692) were amplified. Alignments for *poama-1* showed that a nucleotide mutation at position 333 (G to A) of the *pocama-1* gene without changing the amino acid and *powama-1* did not reveal any variation compared with the GH01 reference strain. On the basis of the low level of genetic diversity detailed in the previous section, we performed a phylogenetic analysis using *ama-1* gene sequences from human, nonhuman primate, avian, and murine malarial species using the NJ method. The results showed that *pocama-1* and *powama-1* occupied a distinct bifurcating branch with 100% bootstrap support (Fig. 1). The *ama-1* gene ID numbers of *Plasmodium* species included in the phylogenetic analysis are provided in additional file 2 (Table S2).

**Fig. 2:**
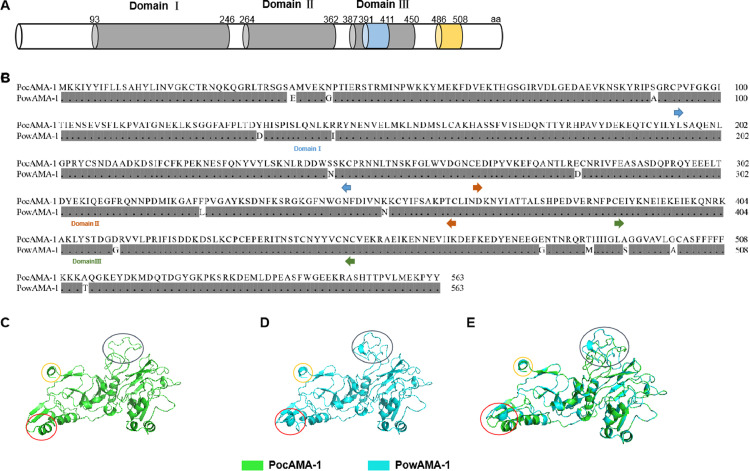
Schematic representation of the protein structure and amino acid sequence alignment of PoAMA-1. (A) Diagram of the protein structure of PoAMA-1. The gray regions are AMA-1 domains; the blue regions are coiled coil domains; the orange regions are transmembrane domains. (B) Amino acid sequences alignment between PocAMA-1 and PowAMA-1. Gray area indicates that the aligned amino acid is identical to the target amino acid. (C) Tertiary structure model of PocAMA-1 protein. (D) Tertiary structure model of PowAMA-1 protein. (E) Tertiary structure model of PoAMA-1 protein. These models are shown in different colors; the PocAMA-1 structure is shown in green, and the PocAMA-1 structure is shown in blue. The tertiary structure of PocAMA-1 and PowAMA-1 mainly has three different areas, which are marked with red, yellow, and black.

The structure of PoAMA-1 protein with 563 amino acids in length was predicted on the basis of a disulfide bond structure and SMART databases. Accordingly, PoAMA-1 contains domain Ⅰ (aa 93–246), domain Ⅱ (aa 264–362), domain Ⅲ (aa 387–450), transmembrane domain (aa 486–508), and a coiled-coil domain (aa 391–411; Fig. 2A) (Hodder et al., 1996).

In addition, the alignment result of the amino acid sequences between *P. ovale curtisi* AMA-1 (PocAMA-1) and *P. ovale wallikeri* AMA-1 (PowAMA-1) showed that PoAMA-1 is highly similar (NCBI BLAST, Similarity 96%); the amino acids between Poc and Pow have 15 difference points (domain Ⅰ: three differences, domain Ⅱ: three differences, domain Ⅲ: one difference, outside the domains: eight differences, Fig. 2B). To obtain a visual comparison between the tertiary structures of PocAMA-1 and PowAMA-1 proteins, the modeling results are displayed in 3D (Fig. 2C, 2D, and 2E). The tertiary structure similarity between PocAMA-1 and PowAMA-1 proteins is also high, and the areas of difference are marked with circles. The identities of *Plasmodium* AMA-1 proteins are 59% (PfAMA-1 was compared with PoAMA-1), 72% (PvAMA-1 was compared with PocAMA-1), and 73% (PvAMA-1 was compared with PowAMA-1) (Additional file 3: Table S3). Moreover, the blast result of E value (Expect) and deletion or insertion (Gaps) is zero (Table S3). The amino acid sequences of PfAMA-1 and PvAMA-1 are similar to PoAMA-1, and the domain regions are marked with different colors of arrows (Additional file 4: Fig. S1A, S1B). Furthermore, the tertiary structures of PfAMA-1 and PvAMA-1 are similar to PoAMA-1 (Fig. S1C, S1D).

### 3.2 Cloning, expression, purification and western blotting analysis of poama-1 with immune serum samples

**Fig. 3:**
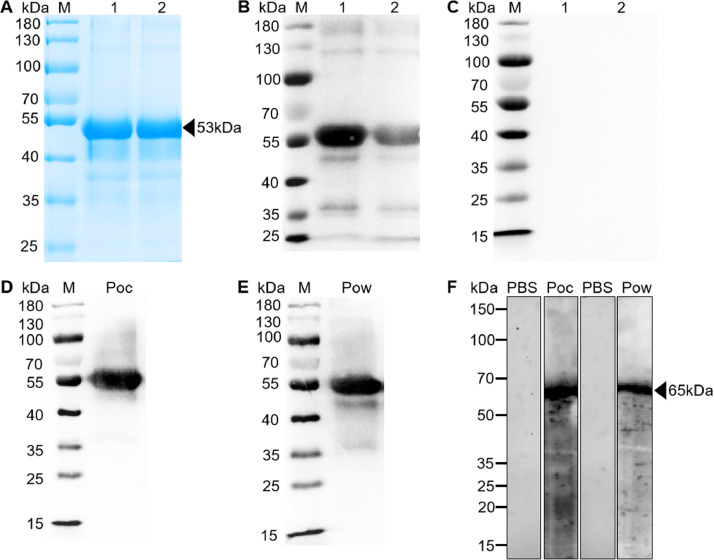
RPoAMA-1 protein expression and anti-rPoAMA-1 antibody detected in rPoAMA-1-immunized mice by SDS-PAGE. (A) Purified rPoAMA-1 (~53 kDa) was resolved by 8% SDS-PAGE. M: protein marker, 1: purified rPocAMA-1, 2: purified rPowAMA-1. (B) Western blot analysis of rPoAMA-1 using anti-His-antibody. M: protein marker, 1: purified rPocAMA-1, 2: purified rPowAMA-1. (C) Western blot analysis of rPocAMA-1 using PBS-immune, rPocAMA-1-immune and rPowAMA-1-immune mouse sera. (D) Western blot showing recognition of native PoAMA-1 antigen in crude proteins from parasite lysate by mouse anti-rPoAMA-1 antibodies (~65 kDa). Arrowheads indicate bands specific to each recombinant protein.

rPoAMA-1 (N-terminal) with a His-tag was expressed using *E. coli* expression system. A single strong target band around 53 kDa demonstrated that the rPoAMA-1 s protein was resolved in 8% SDS-PAGE gel (Fig. 3A, 3B). The concentration of the purified rPoAMA-1 obtained was 1 mg/mL.

The PBS-immunized and rPoAMA-1 immunized mouse sera (Fig. 3C) were evaluated for the antibody specificity of rPoAMA-1-immunized BALB/c mice using specific antibody IgG western blot analysis. These results indicated that rPoAMA-1-immunized mice can produce specific antibodies against rPoAMA-1. Anti-PoAMA-1 antibodies in mice recognized a band of 65 kDa in the crude *P. ovale* antigen, which was extracted from blood stage (Fig. 3D); it showed that anti-PocAMA-1 and anti-PowAMA1 reacted with native AMA-1 in *P. ovale* lysate.

### 3.3 Anti-rPoAMA-1 igg antibody responses and persistence

**Fig. 4:**
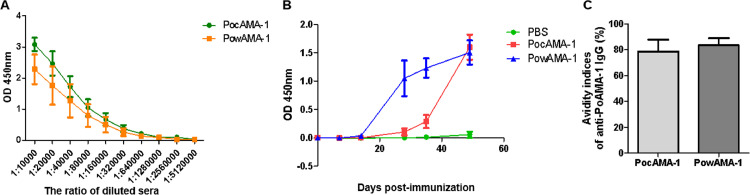
Anti-rPoAMA-1 IgG antibody responses and persistence. (A) Mouse serum IgG titers against rPoAMA-1. Sera obtained from immunized mice were diluted from 1:10,000 to 1:5,120,000, and the data were presented as the mean OD. (B) Days of post immune responses in rPoAMA-1-immunized mice. (C) Avidity indices of anti-PoAMA1 IgG. Significant differences between groups are denoted on the graph: * *P* < 0.05, **P < 0.01, *** *P* <0.001. Nonsignificant (ns) differences are indicated (*P* > 0.05).

To determine the potency of the rPocAMA-1 and rPowAMA-1 proteins, the mice were immunized three times (50 µg/immunization). On all doses tested, rPoAMA-1 s induced a strong antibody response. Following three immunizations with rPoAMA-1, the specific IgG titers of rPoAMA-1-immunized mouse sera were determined by ELISA. The titer against both rPoAMA-1 s was 640,000 (Fig. 4A).

Compared with the PBS-immunized group, no significant anti-rPoAMA-1 IgG antibody was found in the mice immunized for the first time (day 7 and day 14). However, after the first boost (day 28 and 35), the level of anti-rPowAMA-1 IgG antibody increased in all test groups compared with the previous immunization. Following the second booster, the anti-rPocAMA-1 IgG antibody level increased significantly (Fig. 4B), and the IgG antibody levels of the two experimental groups were significantly different from those of the PBS-immunized group (*t-*test, *P* < 0.05).

To evaluate the affinity of the antibody response, the avidity between rPoAMA-1 antigen and IgG antibody in immune mouse was determined by ELISA combined with urea elution. After three immunizations, the anti-rPocAMA-1 and anti-rPowAMA-1 antibodies exhibited high avidity indexes (AI). The average AIs of anti-rPocAMA-1 and anti-rPowAMA-1 IgG were 78.63% and 83.40%, respectively (Fig. 4C).

### 3.4 Cross-reactivity

**Fig. 5:**
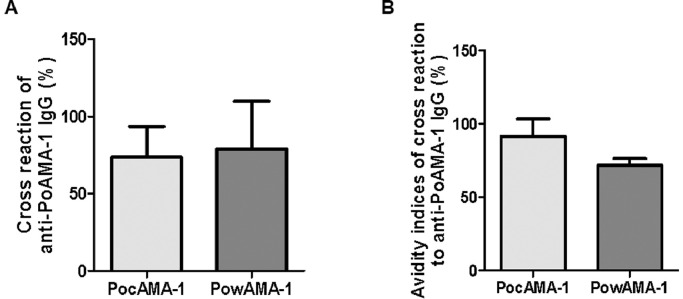
Cross reactivity. (A) Cross reactivity of anti-rPoAMA-1 IgG. (B) Avidity indices of cross reaction to anti-rPoAMA-1 IgG. Significant differences between groups are denoted on the graph: * *P* < 0.05, **P < 0.01, *** *P* <0.001. Nonsignificant (ns) differences are indicated (*P* > 0.05).

The amino acid sequences and the tertiary structures based on protein modeling (Fig. 2B, 2C, 2D, 2E) of PocAMA-1 and PowAMA-1 are similar. Thus, we used ELISA to investigate the cross-reactivity between rPocAMA-1 and rPowAMA-1. The mouse antiserum against rPowAMA-1 reacted with rPocAMA-1 (73.74%, Fig. 5A), and the mouse antiserum raised against rPocAMA-1 was cross-reactive with rPowAMA-1 (79.00%, Fig. 5A). In addition, the affinity of anti-rPocAMA-1 IgG and rPowAMA-1 was 71.69%, and the affinity of anti-rPowAMA-1 IgG and rPocAMA-1 was 91.53% (Fig. 5B). The observed cross-reactivity of PoAMA-1 suggests that a single ELISA could be used to diagnose *P. ovale curtisi* or *P. ovale wallikeri* patients.

### 3.5 Cellular immune response to rPoAMA-1

**Fig. 6:**
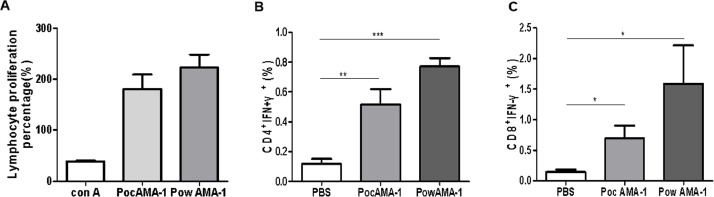
Cellular immune responses of the mice to rPoAMA-1. (A) Lymphocyte proliferation in rPoAMA-1-immunized BALB/c mice and concanavalin A (Con A) as positive control. (B)(C) Measurement of CD4^+^ INF-γ^+^ and CD8^+^ INF-γ^+^levels in mice by flow cytometry. T cells after stimulation with rPoAMA-1; values represent the percentage of either CD4+ or CD8+ *T* cells positive for the individual cytokine. A *t-test* was used to determine differences in production of IFN-γ in response to a stimulus with corresponding antigens. Significant differences between groups are denoted on the graph: * *P* < 0.05, **P < 0.01, *** *P* <0.001. Nonsignificant (ns) differences are indicated (*P* > 0.05).

A proliferation assay was performed to assess the response to rPoAMA-1 proteins *in vitro*. The splenocyte proliferative response against rPoAMA-1-immunized groups was significantly increased compared with the PBS-immunized group (Fig. 6A). The cytokine secretion profiles (IFN-γ) of mice immunized with rPoAMA-1 were determined by intracellular cytokine staining, and the results were analyzed by FACS. The CD4-IFN-γ and CD8-IFN-γ levels in the rPoAMA-1-immunized mice group were significantly higher than those in the PBS-immunized mice group (*P* < 0.05; Fig. 6B, 6C). Splenocytes of rPoAMA-1-immunized mice produced IFN-γ (CD4 and CD8 T cell) after stimulation.

## 4 Discussion

AMA-1 is a micronemal protein, which is essential during the invasion of host cells. However, the variation of *ama-1* is a main obstacle to the study of AMA-1 as a malaria vaccine antigen. *P. vivax* and *P. falciparum* AMA-1 are highly polymorphic molecules (Escalante et al., 2001; Kang et al., 2015). AMA-1 polymorphism in human malaria parasite population has impacted the development of malaria vaccine based on AMA-1 (Hodder et al., 2001; Malkin et al., 2005). We analyzed 26 imported cases of *P. ovale* clinical isolates from Africa to China. We demonstrated that the gene sequences of *powama-1* are completely conserved within our sample set. *pocama-1* within our sample set exhibits low nucleotide diversity, including a nucleotide synonymous mutation. Antigen diversity within the *Plasmodium* is a main factor that may limit the effectiveness of any asexual stage vaccine, including that one based on AMA-1 (Cortés et al., 2003). In addition, the lack of diversity is interesting given that these species have been circulating in Africa for a long time, and patients are from different geographical origins, which may be related to the low transmission rate of *P. ovale* from different geographical origins (Kawamoto et al., 1999).

Antibodies play essential roles in protecting against blood-stage malaria (Hill et al., 2017; Tran et al., 2012; Loiseau et al., 2020). High-affinity antibodies are important for preventing malaria, and affinity is the interaction of a single antibody binding site with an antigenic determinant, which is an essential feature and an important qualitative parameter for an antimalarial immune response (Ferreira et al., 1996). We found that antigen-specific antibodies induced comparable titers (1: 640,000) and high avidities (PocAMA-1: 78.63%, PowAMA-1: 83.40%). Antibodies also recognized native proteins from blood stages, namely, crude antigen. The affinity of antibodies has been associated with increased inhibition of parasite growth as demonstrated in preclinical studies of *P. falciparum* merozoite surface protein 3 (MSP-3) (Druilhe et al., 2005). Interestingly, we found that the amino acid sequences and protein tertiary structure of PfAMA-1, PvAMA-1, and PoAMA-1 are similar. This finding indicated that the AMA-1 protein from different malaria species may have similar functions.

The analysis of PvAMA-1 characteristics using bioinformatics tools for vaccine design showed that several potential B and T cell epitopes were found in PvAMA-1, suggesting that PvAMA-1 would be an effective vaccine target (Jahangiri et al., 2019). Studies have shown that the pentavalent *P. falciparum* AMA-1 vaccine can induce protection against most malaria (Miura et al., 2013). Currently, a potential method to solve the problem of pathogen diversity is to use mixed natural allelic proteins, but the cost of producing complex multivalent antimalaria vaccines is prohibitive (Ouattara et al., 2015). The anti-PocAMA-1 and anti-PowAMA-1 IgG cross-reacted with each other and showed similar antibody responses. These results suggest that rPoAMA-1 has conserved and similar antigenic determinants and that both molecules are highly homologous. These cross-reactivity results indicated that rPoAMA-1 shares similar and conserved antigenic determinants and that the immunity against PoAMA-1 may protect against *P. ovale wallikeri* and *P. ovale curtisi*. The finding suggested that rPoAMA-1 may have similar antigenic determinants that could enable measurement efficacy of species-specific in vaccine trials.

Passive transfer of IgG into animals induced protective immunity against AMA-1, and antibodies against recombinant *P. falciparum* AMA-1 also inhibited invasion of parasite *in vitro* (Hodder et al., 2001). These data suggest that malaria protection was most likely mediated by antibody. However, Pombo et al. demonstrated that *P. falciparum*-protected individuals had a proliferative T cell response involving CD4+ and CD8+ cells; it is a cytokine response composed of interferon gamma (Pombo et al., 2002). The contribution of CD4+ and CD8+ *T* lymphocytes to acquired immunity to blood-stage was confirmed in the study of *P. chabaudi* (Podoba et al. 1991). In summary, immunity to malaria depended on humoral and cellular immune responses (Marsh et al., 2006; Petritus et al., 2008; Roestenberg et al., 2009; Loiseau et al., 2020). The evaluation of cellular immunogenicity showed that the level of CD4+ and CD8+ *T* cells secreting IFN-γ in response to PoAMA-1 protein was very high. Based on the reports on the importance of humoral and cellular immune responses, these results are encouraging for evaluating the immunogenicity of PoAMA-1. Therefore, PoAMA-1 deserves a further study as potential vaccine candidate.

## 5 Conclusions

This study demonstrated that the *poama-1* sequences from *P. ovale curtisi* and *P. ovale wallikeri* parasites isolated from imported cases in the Jiangsu Province were highly conserved and rPoAMA-1 proteins can induce high immunogenicity. The cross-reactivity between rPocAMA-1 and rPowAMA-1 indicated that the immune response to *P. ovale* includes antibodies against shared similar antigenic determinants of both *P. ovale* spp. These results provided valuable reference information on *P. ovale wallikeri* and *P. ovale curtisi* isolates imported from Africa to China.

## Authors’ contributions

**Yang Cheng, Dengxin Zhang and Jun Cao:** Conceived this study, Assisted in writing the manuscript.

**Yao Lei:** Designed the research protocol, Conducted the laboratory work, Data analysis, Wrote the manuscript.

**Feihu Shen:** Conducted the laboratory work, Performed the acquisition of data, Data analysis.

**Haimeng Zhu:** Checked the grammar and spelling of the manuscript, Data analysis.

**Laicheng Zhu:** Collected the samples.

**Ruilin Chu:** Conducted the laboratory work.

**Jianxia Tang and Wenxi Yao:** Data handling and analysis, Reviewed the manuscript.

**Guoding Zhu:** Contributed to interpret the results, Assisted in writing the manuscript.

All authors read and approved the final version of the manuscript.

## Ethics approval and consent to participate

This study was approved by the Ethics Committee, Jiangsu Provincial Key Laboratory on Parasite and Vector Control Technology, Jiangsu Institute of Parasitic Diseases (JIPD) (IRB00004221), Wuxi, China. Informed consent was obtained from all of the participants, and the animal trial was approved by the Animal Ethics Committee, Jiangnan University (JN. No20180615t0900930[100]).

## Funding

This work was supported by the 10.13039/501100001809National Natural Science Foundation of China [81601787, 81871681]; the Natural Science Foundation of Jiangsu Province [BK20160192, BK20150001]; the Jiangsu Provincial Department of Science and Technology [No. BM2018020]; the Jiangnan University Student Innovation Training Program (1285210232175260); the Postgraduate Research & Practice Innovation Program of Jiangnan University (JNKY19_073); the Fundamental Research Funds for the Central Universities funded by the 10.13039/100009950Ministry of Education of China (JUSRP51710A); the Bill & Melinda Gates Foundation (OPP1161962); the National First-class Discipline Program Of Food Science and Technology (JUFSTR20180101), and the Jiangsu Provincial Project of Invigorating Health Care through Science, Technology and Education.

## Declaration of Competing Interest

The authors declare they have no competing interests.

## Appendix Supplementary materials

Image, application 1

Image, application 2

Image, application 3

Image, application 4

Additional files **Additional file 1: Table S1** Country of origin of the *P. ovale curtisi* (14 cases) and *P. ovale wallikeri* (12 cases). **Additional file 2: Table S2***ama-1* Gene ID numbers of other *Plasmodium* species. **Additional file 3: Table S3** Sequence identities of AMA-1 proteins (PfAMA-1, PvAMA-1, PocAMA-1, and PowAMA-1). **Additional file 4: Fig. S1** (A) Amino acid sequence alignment between PfAMA-1 and PoAMA-1. (B) Amino acid sequence alignment between PfAMA-1 and PoAMA-1. (C)(D) Tertiary structure model of PfAMA-1 and PvAMA-1 proteins. Image, application 5
