# Supplementary material for: Low genetic diversity and strong immunogenicity within the apical membrane antigen-1 of plasmodium ovale spp. imported from africa to china
Source: Acta Trop. 2020 Oct;210:105591. doi: 10.1016/j.actatropica.2020.105591 (PMC7456792; doi:10.1016/j.actatropica.2020.105591)
Supplement: Supplementary file 2 [file mmc2.pdf]

Table S1 The country of origin of the *P. ovale curtisi* and *P. ovale wallikeri*.

Species confirmation	Isolate number	Country of origin	Parasitaemia
P. ovale curtisi	Poc-2	Angola	868
	Poc-20	Equatorial Guinea	3124
	Poc-21	Equatorial Guinea	3477
	Poc-22	Equatorial Guinea	7663
	Poc-27	Equatorial Guinea	8834
	Poc-30	Equatorial Guinea	1263
	Poc-34	Equatorial Guinea	11025
	Poc-48	Republic of the Congo	1378
	Poc-62	Cameroon	2736
	Poc-71	Niger	2500
	Poc-77	Nigeria	1013
	Poc-79	Nigeria	1356
	Poc-80	Nigeria	2512
	Poc-87	Unknown	2987
P. ovale wallikeri	Pow-2	Angola	3482
	Pow-3	Angola	10971
	Pow-6	Angola	3354
	Pow-7	Angola	34793
	Pow-8	Angola	20029
	Pow-10	Angola	1024
	Pow-14	Angola	1837
	Pow-23	Equatorial Guinea	9339
	Pow-25	Equatorial Guinea	1829
	Pow-26	Equatorial Guinea	645
	Pow-54	Republic of the Congo	32592
	Pow-89	Sierra Leone	6151
