# Supplementary material for: Low genetic diversity and strong immunogenicity within the apical membrane antigen-1 of plasmodium ovale spp. imported from africa to china
Source: Acta Trop. 2020 Oct;210:105591. doi: 10.1016/j.actatropica.2020.105591 (PMC7456792; doi:10.1016/j.actatropica.2020.105591)
Supplement: Supplementary file 3 [file mmc3.pdf]

Table S2 The *ama-1* Gene ID number of other *Plasmodium* species

Species	Strain	Gene ID from PlasmoDB
P. falciparum	3D7	PF3D7_1133400
	IT	PfIT_110038000
P. praefalciparum	strain G01	PPRFG01_1134200
P. reichenowi	CDC	PRCDC_1131800
	G01	PRG01_1130800
P. gaboni	strain G01	PGABG01 _1131400
	strain SY75	PGSY75_1133400
P. vinckei petteri	strain CR	YYG_01961
P. vinckei vinckei	strain vinckei	YYE_03007
P. berghei	ANKA	PBANKA_0915000
P. yoelii	yoelii 17XNL	PY01581
	yoelii YM	PYYM_0916000
P. gallinaceum	8A	PGAL8A_00360900
P. malariae	UG01	PmUG01_09042600
P. knowlesi	strain H	PKNH_0931500
	strain Malayan Strain Pk1 A	PKNOH_S120150200
P. inui	San Antonio 1	C922_ 02330
P. cynomolgi	strain B	PCYB_093930
	strain M	PcyM_0938200
P. vivax	P01	PVP01_0934200
	Sal-1	PVX_092275
