# Supplementary material for: Low genetic diversity and strong immunogenicity within the apical membrane antigen-1 of plasmodium ovale spp. imported from africa to china
Source: Acta Trop. 2020 Oct;210:105591. doi: 10.1016/j.actatropica.2020.105591 (PMC7456792; doi:10.1016/j.actatropica.2020.105591)
Supplement: Supplementary file 4 [file mmc4.pdf]

Table S3 The identities of AMA-1 proteins

AMA-1	PfAMA-1	PvAMA-1
PocAMA-1	59%	72%
PowAMA-1	59%	73%
