# Supplementary material for: Low genetic diversity and strong immunogenicity within the apical membrane antigen-1 of plasmodium ovale spp. imported from africa to china
Source: Acta Trop. 2020 Oct;210:105591. doi: 10.1016/j.actatropica.2020.105591 (PMC7456792; doi:10.1016/j.actatropica.2020.105591)

**Additional file 4: Fig. S1** (A) Amino acid sequence alignment between PfAMA-1 and PoAMA-1. (B) Amino acid sequence alignment between PfAMA-1 and PoAMA-1. (C)(D) Tertiary structure model of PfAMA-1 and PvAMA-1 proteins.


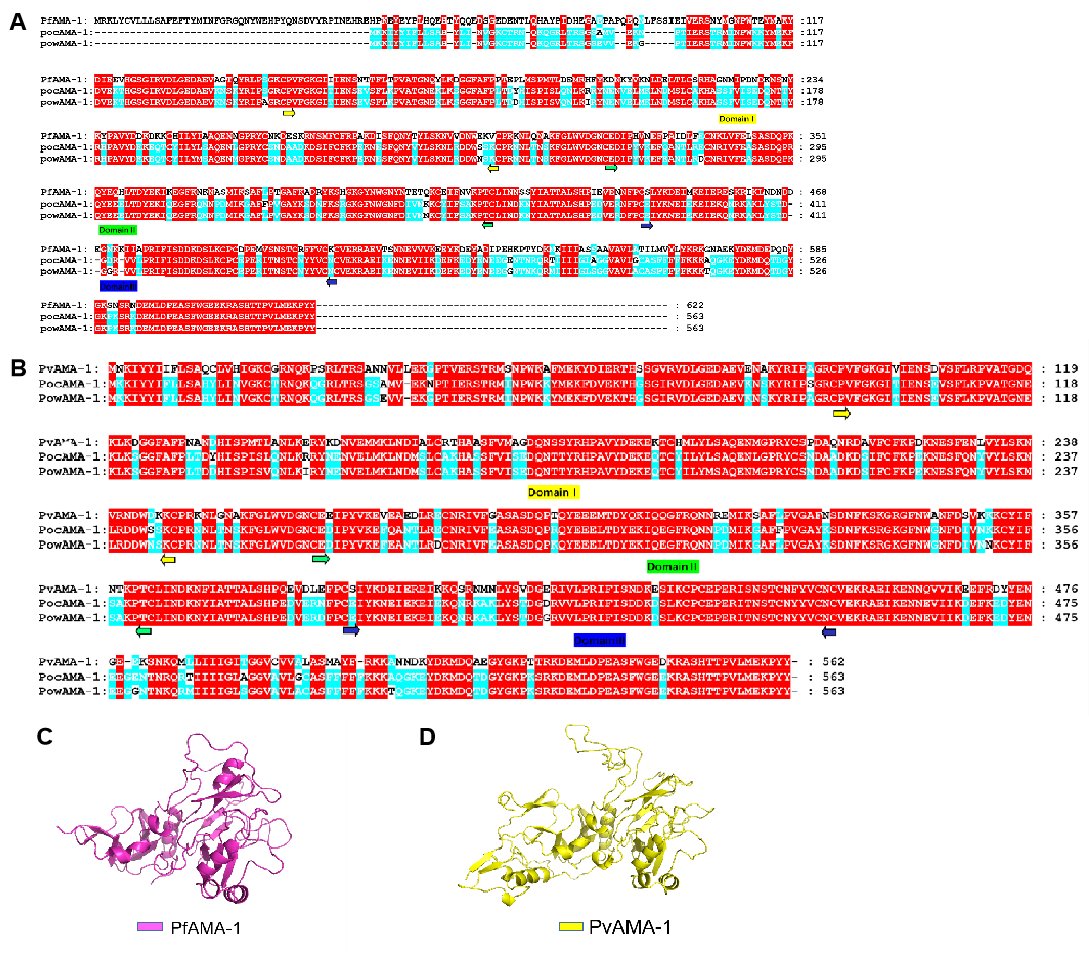

Supplement: Supplementary file 5 — Additional files Additional file 1: Table S1 Country of origin of the P. ovale curtisi (14 cases) and P. ovale wallikeri (12 cases). Additional file 2: Table S2ama-1 Gene ID numbers of other Plasmodium species. Additional file 3: Table S3 Sequence identities of AMA-1 proteins (PfAMA-1, PvAMA-1, PocAMA-1, and PowAMA-1). Additional file 4: Fig. S1 (A) Amino acid sequence alignment between PfAMA-1 and PoAMA-1. (B) Amino acid sequence alignment between PfAMA-1 and PoAMA-1. (C)(D) Tertiary structure model of PfAMA-1 and PvAMA-1 proteins. [file mmc5.docx]
